# Relationships between depression and anxiety symptoms and adipocyte-derived proteins in postmenopausal women

**DOI:** 10.1371/journal.pone.0248314

**Published:** 2021-03-05

**Authors:** Yu-Ting Wu, Wan-Yu Huang, Chew-Teng Kor, Ko-Hung Liu, Ting-Yu Chen, Po-Te Lin, Hung-Ming Wu

**Affiliations:** 1 Center for Mitochondrial Medicine and Free Radical Research, Changhua Christian Hospital, Changhua, Taiwan; 2 Pediatrics of Kung-Ten General Hospital, Taichung City, Taiwan; 3 Internal Medicine Research Center, Changhua Christian Hospital, Changhua, Taiwan; 4 Inflammation Research & Drug Development Center, Changhua Christian Hospital, Changhua, Taiwan; 5 Department of Neurology, Changhua Christian Hospital, Changhua, Taiwan; 6 Graduate Institute of Acupuncture Science, China Medical University, Taichung, Taiwan; Universidad Nacional Autonoma de Mexico, MEXICO

## Abstract

**Introduction:**

Studies on the association between adiponectin and leptin and anxiety and depression among postmenopausal women are limited. Therefore, the present study specifically evaluates the mutual relationships between adiponectin and leptin and anxiety and depression in postmenopausal women.

**Participants and design:**

In this cross-sectional study, a total of 190 women aged 40–65 years were enrolled. Depression symptoms were assessed using the Center for Epidemiologic Studies Depression Scale (CES-D), and anxiety symptoms were evaluated using the Hamilton Anxiety Rating Scale (HAM-A). Fasting specimens were collected to measure sex hormone, glucose, insulin, and adipokine levels. Multiple linear regression analysis was performed to evaluate the associations between depression and anxiety and adipocyte-derived hormones.

**Settings:**

The study was performed in a hospital medical center.

**Results:**

Among 190 enrolled postmenopausal women, Spearman’s rank correlation analysis revealed significant correlations between CES-D and HAM-A (r = 0.715, *P* < 0.0001), between CES-D and adiponectin (*p* = 0.009) and leptin (*p* = 0.015), and between HAM-A and adiponectin (*p* = 0.01) and leptin (*p* = 0.001). The subjects with CES-D ≥ 16 and with HAM-A ≥ 18 had higher adiponectin levels than those with CES-D < 16 and HAM-A < 18, respectively. After adjusting for age, body mass index, exercise, alanine amino transferase and parameters of lipid profiles, Log adiponectin levels were found to be significantly associated with both CES-D and HAM-A, and Log leptin levels were only significantly associated with HAM-A.

**Conclusions:**

The data show that adiponectin and leptin levels are significantly associated with depression and anxiety symptoms. These results suggest that higher adiponectin and lower leptin levels may serve as potential markers related to anxiety and mood in postmenopausal women. More future research that is designed to deal with the important confounders (e.g., population heterogeneity) is needed to investigate comprehensively on these associations.

## Introduction

Depression and anxiety are two common health problems among middle-aged women. The risk of these tow mental illness occurrence has been reported to increase in women during the perimenopausal and postmenopausal stages [1–3]. The development of depression and anxiety symptoms is respectively approximately 1.8 and 2.0 times higher in the menopausal period than in the premenopausal period [3–5]. The prevalence of depression and anxiety symptoms among peri- and postmenopausal women is 18%–41.8% and 7%–25%, respectively [2, 3, 5–8]. Although clear and common causative factors have yet to be established, these data suggest a relationship between mental health problems and menopause.

Several studies have shown that depression is closely linked to obesity, metabolic syndrome, and insulin resistance [9, 10]. Everson–Rose et al. reported that depression is associated with higher values of homeostatic model assessment for insulin resistance and incident diabetes in middle-aged women over a 3-year follow-up period [11] and could predict the development of incident type 2 diabetes [12]. Other studies have consistently demonstrated that women with higher levels of depressive symptoms are at higher risk of developing cardiovascular diseases and impaired cognitive function than women with lower levels of depressive symptoms [13]. Compared with studies on depression, research on anxiety among women in midlife is relatively less reported [1, 14]. Jaremkaa and Pacanowskib reported that social anxiety symptoms may interact with obesity [15, 16]. The authors found that patients with obesity and more social anxiety symptoms have greater inflammation and insulin resistance than those with less anxiety symptoms [15]. Narita et al. reported that the severity of anxiety traits is positively associated with insulin resistance [16]. These results suggest that depression and anxiety may be associated with increased risks of developing metabolic and chronic systemic diseases (e.g., cardiovascular disease).

Adipose tissue, which is composed mostly of adipocytes, is considered an important endocrine organ that is closely related to immune and metabolism. Adipocytes primarily secrete two main hormones in humans, namely, adiponectin and leptin. Adiponectin is known to have anti-inflammatory and insulin-sensitizing effects [17, 18]. Previous cross-sectional and longitudinal studies revealed that adiponectin levels are inversely related to obesity and metabolic disorders (e.g., diabetes, insulin resistance, and impaired fasting glucose) [19, 20]. Leptin is a pro-inflammatory factor that is intimately involved in metabolic regulation and energy balance [18]. High leptin levels are associated with increased risks for cardiovascular disorders, such as increased carotid artery intimal–medial thickening [21] and incident ischemic coronary disease in patients with diabetes [22].

The role of adiponectin and leptin in mental illness has gradually gained increased research attention. While previous studies have examined the association of these hormones with anxiety and depression, inconsistent results have been obtained [23, 24]. For example, a meta-analysis revealed that peripheral adiponectin levels in patients with depression are not different from those in the healthy population, although the hormone levels observed revealed some differences among subgroups of subjects with depression compared with controls [23]. Studies on the association between adiponectin and leptin and anxiety and depression among postmenopausal women are limited [25]. Increases in the incidence of chronic systemic diseases (e.g., diabetes) and mental illness have been observed in menopausal women. Therefore, the present study specifically evaluates the mutual relationships between adiponectin and leptin and anxiety and depression in postmenopausal women.

## Subjects and methods

### Participants and study design

Postmenopausal women (age, 40–65 years) who had visited the Changhua Christian Hospital between July 2017 and December 2019 were considered eligible for participation in this cross-sectional study. Postmenopause was defined as over 12 consecutive months of amenorrhea prior to study entry. Women were excluded if they were at the perimenopausal stage, used hormone therapy, or had a medical history of diabetes (defined as fasting blood glucose > 126 mg/ml, postprandial blood glucose > 200 mg/ml, or on antidiabetic medication), mixed hyperlipidemia (defined as total cholesterol > 240 mg/dL, triglycerides > 200 mg/dL, or on statin medication), primary hypertension (defined as blood pressure >140/90 mmHg or on antihypertensive medication), or thyroid disease. In addition, women who were hepatitis B and C carriers and had liver function impairment were also excluded. Written informed consent was obtained from all participants. The present study was approved by the Changhua Christian Hospital Institutional Review Board (No. 150609).

### Anthropometric measurements

Blood specimens were obtained from each participant in the morning after at least an overnight 8-hour fast at study entry. Plasma was separated from the blood specimens, aliquoted, and stored at −80°C until assay. Systolic and diastolic blood pressure was measured for each participant, and height and weight were measured in light clothing without shoes. Body mass index (BMI) was calculated as weight (kg)/[height (m)]^2^.

### Psychological measurements

All participants completed a questionnaire corresponding to the Hamilton Anxiety Rating Scale (HAM-A) [26] and the Center for Epidemiologic Studies Depression Scale (CES-D) to evaluate anxiety and depression traits [27]. HAM-A has been used extensively in research and clinical practice. The HAM-A is composed of 14 specifically formatted items, including anxious mood, tension, fears, difficulty falling sleep, somatic complaints, and behavior at the interview. Each item is scored on a 5-point scale ranging from 0 (not present) to 4 (very severe). The CES-D is a short self-report scale designed to measure depressive symptoms; it is widely applied to screen instruments in primary care clinics and research [27]. The scale is composed of 20 items and used to assess the various symptoms of depression as they occurred in the past week; the majority of these items focus on the affective component of depression.

### Measurement of adipocyte-derived proteins

Plasma levels of adipocyte-derived proteins and insulin were measured as described previously [28]. Briefly, plasma resistin and adiponectin levels were measured using human adipokine multiplex assays (Milliplex MAP kits, EMD Millipore, Billerica, MA, USA), and plasma leptin levels were measured using human metabolic hormone multiplex assays (Milliplex MAP kits, EMD Millipore, Billerica, MA, USA). The data of these three adipocyte-derived proteins were read using a Luminex 200 system with a dynamic range of ≥3.5 log units (Luminex, Austin, TX, USA) and then collected and analyzed using an instrument equipped with MILLIPLEX Analyst software (EMD Millipore, Billerica, MA, USA). Values for leptin and resistin are reported as ng/ml, while that for adiponectin are reported as μg/ml. The lower limits of detection (pg/ml) were as follows: leptin, 135.4; adiponectin, 29.54; and resistin, 4.74. Plasma samples of leptin were assayed undiluted while those of adiponectin and resistin were assayed using a 1:200 dilution in assay buffer. If the measured sample levels were beyond the range of detection, the samples were retested after optimal dilution. All plasma samples were assayed in duplicate. The intra-assay laboratory coefficients of variation (CVs, %) were as follows: leptin, 7.2; resistin, 5.8; and adiponectin, 8.1. All inter-assay CVs of these three proteins were <10%. Two replicates of a control sample were tested on each microplate in any given run.

### Sexual hormone measurements and biochemical determinations

Serum levels of the sex hormones estradiol (E2) and follicle-stimulating hormone (FSH), as well as total cholesterol, triglycerides, low-density lipoprotein cholesterol (LDL-C), high-density lipoprotein cholesterol (HDL-C), fasting glucose, alanine aminotransaminase (GPT), and aspartate aminotransaminase (GOT), were measured using standard procedures at the Department of Laboratory Medicine, Changhua Christian Hospital. In brief, FSH and E2 levels in undiluted specimens were measured using the Access hFSH assay and the Access Estradiol assay on the Beckman Access Immunoassay system with a dynamic range of ≥3.5 log units (Beckman Coulter, Fullerton, CA, USA). The lower limits of detection for E2 and FSH were 20 pg/ml and 0.2 mIU/mL, respectively. All measured specimens were analyzed in duplicate and within the range of detection of the standard curve. The inter- and intra-assay laboratory CVs for E2 were <7.6% and 8.2%, respectively, and the corresponding CVs for FSH were <8.1% and 5.3%, respectively.

### Statistical analysis

Results are presented as medians (interquartile range [IQR]). Kolmogorov-Smirnov test was used to determine whether data fit with the normal or non-normal distribution. Differences between two groups of participants were assessed using the Mann-Whitney U test. Correlations were determined using Spearman’s rank correlation analysis. After logarithmic transformation for adiponectin and leptin levels, associations between CES-D or HAM-A and adipocyte-derived hormones were determined by univariate analysis as well as multivariate linear regression after adjusting for BMI, age, exercise, GPT, and parameters of lipid profiles. Statistical analyses were performed using SPSS version 19.0.0 (IBM Corp., Somers, NY, USA). A two-tailed *p-*value < 0.05 was considered significant.

## Results

### Demographic, clinical, and laboratory data

A total of 190 postmenopausal women who fulfilled the inclusion criteria were enrolled in this study. The women had no medical history of chronic systemic diseases, including diabetes, hypertension, and liver function impairment. Table 1 shows that the median (IQR) values of age, menopause duration, FSH level, and estradiol level in these women are 53 (50,56) years, 2.5 (1,5) years, 64.8 (38.6,80.3) mIU/mL, and <20 pg/ml, respectively. The median values of CES-D and HAM-A were 10.5 (6,16) and 15 (9,21), respectively (Table 1). Clinically significant depressive symptoms (CES-D score ≥ 16) [29] were found in 28.9% of the subjects, and 15.8% of the subjects had moderate to severe depression (score ≥ 21), while anxiety traits (HAM-A score ≥ 18) [30] were observed in 31.1% of the subjects, and 14.7% of the subjects had severe to very severe anxiety (score ≥ 25).

**Table 1:** Basic characteristic of the participants (n = 190).

Variables	Median (IQR) or Number (%)
Age, years	53 (50, 56)
MP duration, years	2.5 (1, 5)
CES-D	10.5 (6, 16)
HAM-A	15 (9, 21)
BMI, kg/m^2^	22.65 (21, 24.8)
FSH, mIU/mL	64.8 (38.6, 80.3)
Estradiol, pg/mL	20 (20, 20)
Fasting glucose, mg/dL	94 (89, 100)
Hemoglobin A1c, %	5.5 (5.3, 5.7)
GOT, U/L	22 (20, 25)
GPT, U/L	19 (16, 24)
Total cholesterol, mg/dL	200 (181, 230)
Triglyceride, mg/dL	92 (64, 129)
HDL-C mg/dL	59 (50, 69)
LDL-C, mg/dL	119 (99, 144.8)
Adiponectin, μg/mL	25.4 (15.1, 44)
Leptin, ng/mL	8.4 (4.7, 14)
Resistin, ng/mL	263 (203, 416)
Leptin-to-adiponectin ratio	0.3 (0.2, 0.8)
Smoking	6 (3.2%)
Alcohol consumption	2 (1.1%)
Exercise	91 (47.9%)

Data are presented as mean ± standard deviation (SD) and median (Q1, Q3).

Abbreviations: Q, quarter; Q1, 25^th^ percentile; Q3, 75^th^ percentile; MP duration, duration of menopause since final menstrual period; CES-D, Center for Epidemiologic Studies Depression Scale; HAM-A, Hamilton Anxiety Rating Scale; FSH, follicle stimulating hormone; BMI, body mass index; HDL-C, high-density lipoprotein cholesterol; LDL-C, low-density lipoprotein cholesterol; GOT, aspartate aminotransferase; GPT, alanine aminotransferase.

### Correlations between CES-D, HAM-A, and adipocyte-derived proteins

**Fig 1:**
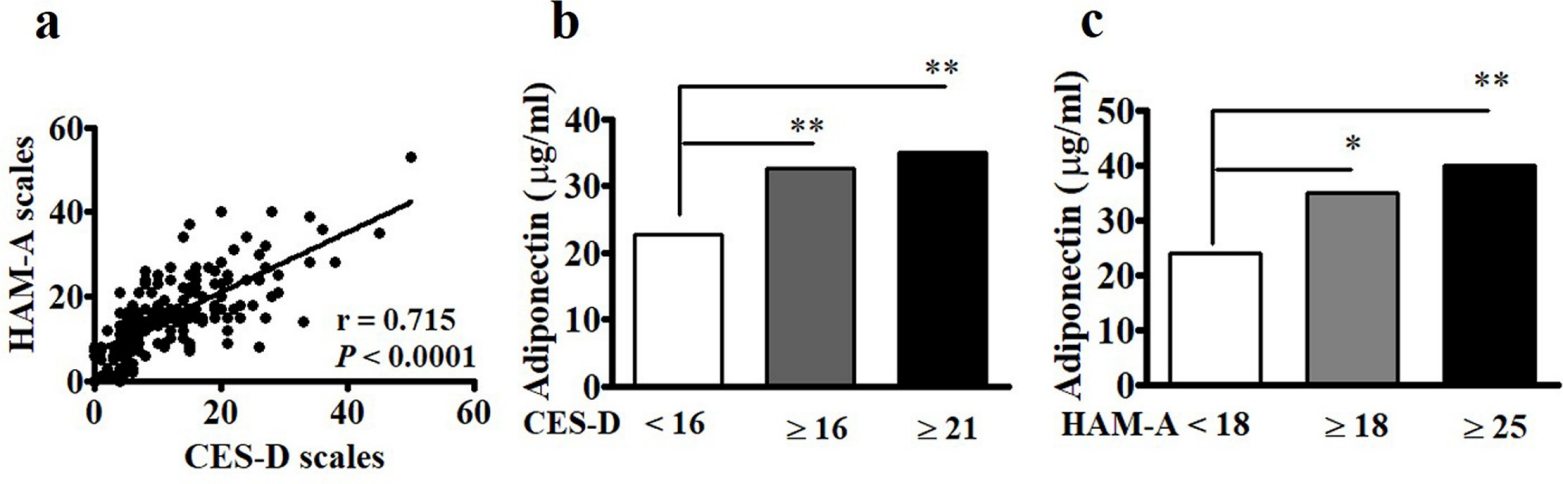
Associations between Center for Epidemiologic Studies Depression Scale (CES-D) and Hamilton Anxiety Rating Scale (HAM-A) and adiponectin levels. (a) CES-D was significantly correlated with HAM-A. Statistical analysis was conducted via Spearman’s rank analysis. (b and c) The levels of adiponectin in subjects with CES-D ≥ 16 and CES-D ≥ 21 and HAM-A ≥18 and HAM-A ≥ 25 were significantly higher than those with CES-D <16 and HAM-A < 18, respectively. Mann-Whitney U test was conducted to compare the medians between two populations. *, *p* < 0.05; **, *p* < 0.001.

**Fig 2:**
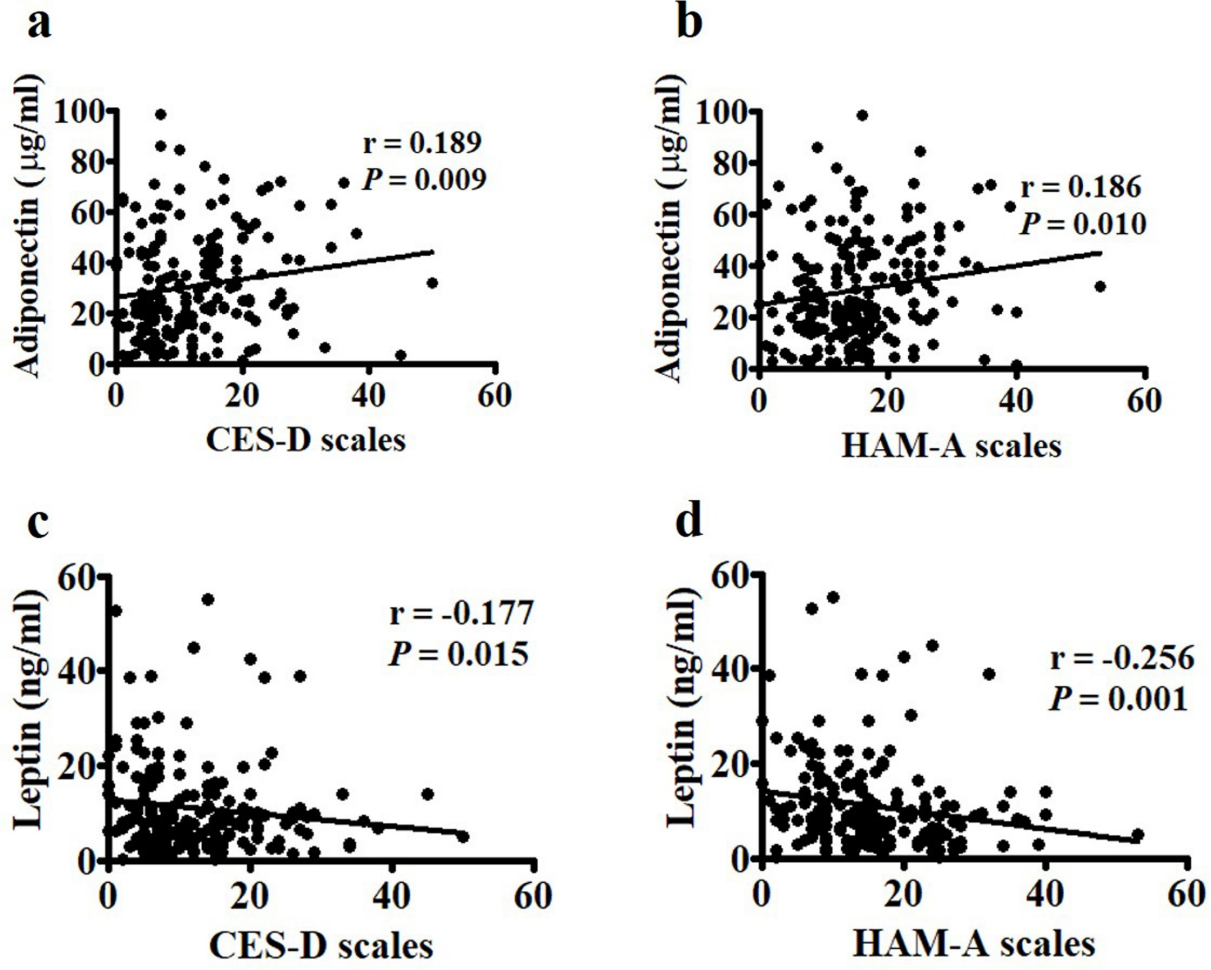
Correlations between Center for Epidemiologic Studies Depression Scale (CES-D), Hamilton Anxiety Rating Scale (HAM-A), and adipocyte-derived proteins. (a and b) CES-D and HAM-A were positively correlated with adiponectin and (c and d) negatively correlated with leptin. Statistical analysis was conducted via Spearman’s rank analysis.

Although each condition has its own causes, depression and anxiety can occur simultaneously. Spearman’s correlation analysis revealed significant associations between the CES-D and HAM-A scales (r = 0.715, *p* < 0.0001) among the postmenopausal women in this study (Fig 1A). The median levels of adiponectin in cases with either CES-D ≥ 16 and CES-D ≥ 21 were higher than those in cases with CES-D < 16 (Fig 1B). Similarly, the median values of adiponectin in subjects with either HAM-A ≥ 18 and HAM-A ≥ 25 were higher than those in those with HAM-A < 18 (Fig 1C). We further found significantly positive associations between CES-D and HAM-A and adiponectin (Fig 2A and 2B) and negative associations between CES-D and HAM-A and leptin (Fig 2C and 2D).

### Multivariate linear regression analyses of the variables associated with CES-D and HAM-A

Univariate analysis revealed that Log adiponectin levels are significantly associated with BMI (r = -0.161, *p* = 0.027), GPT (r = -0.185, *p* = 0.011), and HDL-C (r = 0.244, *p* = 0.001) as well as marginally significantly with CES-D (r = 0.135, *p* = 0.062) and HAM-A (r = 0.136, *p* = 0.060) (S1 Table). Log leptin levels are significantly associated with BMI (r = 0.326, P < 0.001), HDL-C (r = -0.207, *p* = 0.005), GPT (r = 0.148, *p* = 0.041) and HAM-A (r = -0.175, *p* = 0.016) (S1 Table). Furthermore, multivariate linear regression analysis was used to identify the most important factors influencing the relationships between Log adiponectin, Log leptin, and these two mental symptoms. After adjusting for age, exercise, BMI, GPT, TG, HDL-C, and LDL-C, Log adiponectin levels are found to be significantly associated with both CES-D (*p* = 0.034) and HAM-A (*p* = 0.045) (Table 2), and Log leptin levels are only significantly associated with HAM-A (*p* = 0.020) (Table 3).

**Table 2:** Multivariate analysis associations of adiponectin levels with CES-D, and HAM-A.

Variables	Log (adiponectin)					
	Adj. Beta (95% CI)	Adj. r	*p*-value	Adj. Beta(95% CI)	Adj. r	*p*-value
CES-D	0.015(0.001,0.029)	0.156	0.034	--	--	--
HAM-A	--	--	--	0.014(0.0004,0.028)	0.145	0.045
BMI	-0.022(-0.063,0.018)	-0.082	0.284	-0.023(-0.063,0.018)	-0.084	0.271
HDL-C	0.043(0.009,0.076)	0.698	0.013	0.040(0.007,0.073)	0.654	0.018
GPT	-0.014(-0.032,0.003)	-0.123	0.100	-0.015(-0.033,0.002)	-0.132	0.078
TG	0.006(0.000,0.012)	0.468	0.069	0.005(-0.001,0.012)	0.423	0.096
LDL-C	0.027(-0.005,0.060)	1.123	0.096	0.025(-0.007,0.057)	1.028	0.125

Adjusted for age, BMI, exercise, HDL-C, GPT, TG, and LDL-C.

Abbreviations: Adj., Adjusted; CES-D, Center for Epidemiologic Studies Depression Scale; HAM-A, Hamilton Anxiety Rating Scale; BMI, body mass index; HDL, high-density lipoprotein cholesterol; GPT, alanine amino transferase; TG, triglyceride; LDL, low-density lipoprotein cholesterol.

**Table 3:** Multivariate analysis associations of leptin levels with CES-D, and HAM-A.

Variables	Log (leptin)					
	Adj. Beta (95% CI)	Adj. r	*p*-value	Adj. Beta (95% CI)	Adj. r	*p*-value
CES-D	-0.01(-0.024,0.004)	-0.101	0.152			
HAM-A	--	--	--	-0.017(-0.03,-0.003)	-0.162	0.020
BMI	0.068(0.028,0.109)	0.244	0.001	0.067(0.027,0.107)	0.241	0.001
HDL-C	-0.006(-0.016,0.003)	-0.101	0.206	-0.006(-0.016,0.003)	-0.100	0.205
GPT	0.01(-0.007,0.027)	0.080	0.263	0.01(-0.007,0.027)	0.083	0.242
TG	0(-0.002,0.002)	0.031	0.684	0(-0.001,0.002)	0.036	0.628
LDL-C	0.003(-0.001,0.006)	0.112	0.119	0.003(0,0.007)	0.122	0.087

Adjusted for age, BMI, exercise, HDL-C, GPT, TG, and LDL-C.

Abbreviations: Adj., Adjusted; CES-D, Center for Epidemiologic Studies Depression Scale; HAM-A, Hamilton Anxiety Rating Scale; BMI, body mass index; HDL-C, high-density lipoprotein cholesterol; GPT, alanine amino transferase; TG, triglyceride; LDL-C, low-density lipoprotein cholesterol.

## Discussion

Investigating the associations between depression and anxiety and adipocyte-derived hormones provides insights into the role of adipose tissues in mental disorders in postmenopausal women. CES-D and HAM-A scores were significantly positively correlated with adiponectin and negatively correlated with leptin levels in this population. The subjects with significant depressive symptoms (e.g., CES-D ≥ 16 and CES-D ≥ 21) and with significant anxiety symptoms (e.g., HAM-A ≥ 18 and HAM-A ≥ 25) had higher adiponectin levels than those without depression and anxiety (i.e., CES-D < 16 and with HAM-A < 18) (Fig 1B and 1C). After adjusting for age, exercise, BMI, and parameters of lipid profiles, these associations remained significant, thus suggesting that circulating Log adiponectin and Log leptin levels may be linked to depression and anxiety symptoms in postmenopausal women.

The prevalence of mood disorders, such as depression and anxiety, is inconsistent and varies greatly [7] but generally ranges from 12% to 50% among women, especially during menopause [31]. Similar to previous reports [32], approximately a quarter of the postmenopausal women in the present study experienced depression traits of CES-D ≥ 16 and/or anxiety traits of HAM-A ≥ 18; moreover, about 15% of the surveyed women had moderate to severe depression and/or severe to very severe anxiety. In fact, depression and anxiety are two different but commonly co-existing mental health disorders. Our study consistently found that CES-D is highly correlated with HAM-A (Fig 1A) [4].

Adipose tissue is considered a dynamic endocrine organ that essentially regulates glucose and lipid metabolism in humans. For instance, adiponectin is involved in activities of cholesterol efflux, influencing overall reverse cholesterol transport and subsequent increasing plasma HDL-C levels [33, 34]. Leptin particulates in cholesterol-regulating functions via proprotein convertase subtilisin/kexin type 9 pathway and down-regulates expression of LDL receptors in hepatocytes, leading to elevated plasma LDL-C levels and dyslipidemia [35]. High levels of HDL-C can lower your risk for cardiovascular diseases, while high LDL-C levels have opposite effects [36, 37]. Among our postmenopausal participants, we observed the findings that circulating adiponectin levels are significantly positively associated with HDL-C levels (Table 2) and leptin levels are highly correlated with LDL-C (Table 3), consistent with previous reports [38, 39]. The results suggest these two adipocyte-derived proteins could act as the markers predicting with risk for cardiovascular diseases in postmenopausal women. Recent evidence shows that adiponectin and leptin can enter the brain through the peripheral circulation to exert a wide range of biological functions, such as neuroprotection and mood and mental regulation [40–43]. Several animal studies have demonstrated that adiponectin-deficient mice exhibit increased susceptibility to depression-related (e.g., social interaction) and anxiety-related behaviors as well as other stress-related disorders [41, 44]. Leptin has also been found to have an important function in regulating moods [42, 43].

An earlier meta-analysis illustrated a lower peripheral level of adiponectin in depressed patients compared with that in controls and suggested that males who have lower adiponectin and leptin levels have an increased likelihood of developing major depression [45]. Vuong and colleagues also found an inverse association between adiponectin levels and anxiety disorder [25]. Other meta-analysis reports have shown that the associations of adiponectin levels with depression are not sufficiently clear in different populations, although low adiponectin levels have been observed in the European subgroup [26, 27]. The initial enthusiasm regarding the possible utility of adiponectin and leptin as diagnostic depression biomarkers has not been justified [26]. Clinical studies on the possible association of leptin levels with anxiety and depression are relatively limited and divergent [46, 47]. Some studies indicate that patients with major depression have low leptin levels in the plasma and cerebrospinal fluid [46]. However, another clinical study reported that plasma leptin levels do not differ between depressed patients and healthy controls [47].

These results seem inconsistent and paradoxical but probably arise from individual research limitations, including heterogeneity, sample size, and potential publication bias, BMI, depression severity, and heterogeneity of depressive disorders, and differences in assay types for adiponectin and leptin [23–25, 45]. Moreover, depression and anxiety are often comorbid with subclinical metabolic and cardiovascular disorders [48, 49]. A large cross-sectional study reported a correlation between reduced adiponectin levels in early stages of diabetes or prediabetes and the severity of depression [50], that is, underlying metabolic disorders represent an initial confounding factor for adipokine-related studies. In addition, increasing reports have shown that liver function impairment and steatosis are strongly correlated with adiponectin in the patients with viral hepatitis (e.g., hepatitis C virus) [51, 52] and non-alcoholic steatohepatitis [53]. The present study has been designed to deal with these important confounders that are frequently seen in postmenopausal women. Our study may therefore provide a relatively reliable way of assessing the relationships between mental illness and adipocyte-derived proteins.

The possible associations of adipocyte-derived hormones with depression and anxiety may vary among special populations. Yildiz and colleagues reported that serum levels of adiponectin and leptin were high in women with postpartum depression [54]. In a 5-year follow-up, Dae Jong Oh and colleagues found that higher plasma adiponectin levels may precede the development of clinically significant depression in the elderly in a community-dwelling [55]. Adam and colleagues reported that low mood may be significantly related to adiponectin and BMI in premenopausal women but not in postmenopausal women [56]. Everson–Rose and colleagues reported that the severity of depressive symptoms is significantly associated with lower levels of adiponectin among healthy middle-aged women in both cross-sectional and 5-year follow-up longitudinal analyses [57]. Although in this analyses, these factors age, adiposity, hormonal and behavioral risk factors (e.g., diet, smoking status, alcohol consumption, and physical exercise), all of which are potentially important confounders have been taken into consideration, the levels of the adiponectin and leptin were highly changed and varied by menopause stages in women at midlife with/without obesity [58]. Our data showed that CES-D and HAM-A are positively associated with plasma adiponectin levels but negatively associated with plasma leptin levels. While the postmenopausal population in the present study was relatively homogeneous and healthy, this result is not completely consistent with the findings observed from animal experiments [43–46]. Depression during menopause has a multifactorial etiology. The important contributors to this mental illness include previous depressive episodes, comorbidities with menopausal symptoms, such as vasomotor symptoms, sleep problems, stressful life events, body weight gain, low social support, and ethnicity [41, 59]. These associated factors may affect the expression of adipokines such as adiponectin, leptin, and inflammatory factors. Thus, considering the underlying factors is necessary in future studies to evaluate the relationships between depression and anxiety and adipocyte-derived proteins comprehensively.

There are several limitations to the present study that need to be addressed. First, this cross-sectional designed investigation did not allow us to determine whether a causal relationship exists between depression and anxiety symptoms, and adipocyte-derived hormones. Second, the number (n = 190) of postmenopausal women in the present study was relatively small, although there were significant associations of CES-D and HAM-A with adipocyte-derived proteins. However, our postmenopausal population was relatively homogeneous without systemic and underlying diseases. Therefore, the results may provide more reliable evidence for associations between these mental illnesses and adipocyte-derived proteins, but not be applicable to other populations (e.g. diabetes groups).

In conclusion, our findings show that depression and anxiety symptoms are significantly associated with circulating adiponectin and leptin levels in postmenopausal women. These results suggest that higher adiponectin levels and lower leptin levels may serve as the potential markers related to clinically significant depression symptoms (e.g., CES-D ≥ 16 and CES-D ≥ 21) and anxiety symptoms (e.g., HAM-A ≥ 18 and HAM-A ≥ 25) in postmenopausal population. In future research, more studies that are designed to deal with the important confounders (e.g., population heterogeneity and heterogeneity of depression and anxiety disorders) are needed to investigate comprehensively on the relationships between these two mental symptoms and adipocyte-derived proteins.

## Supporting information

S1 Table Univariate analysis for associations of adiponectin and leptin levels with other parameters. (DOCX)
