# Supplementary material for: Relationships between depression and anxiety symptoms and adipocyte-derived proteins in postmenopausal women
Source: PLoS One. 2021 Mar 5;16(3):e0248314. doi: 10.1371/journal.pone.0248314 (PMC7935290; doi:10.1371/journal.pone.0248314)
Supplement: S1 Table — (DOCX) [file pone.0248314.s001.docx]

**S1 Table. Univariate analysis for associations of adiponectin and leptin levels with other parameters**

| **Variables** | **Log (adiponectin)** | | **Log (leptin)** | |
| --- | --- | --- | --- | --- |
|  | **r** | ***p*-value** | **r** | ***p*-value** |
| CES-D | 0.135 | 0.062 | -0.106 | 0.144 |
| HAM-A | 0.136 | 0.060 | -0.175 | 0.016 |
| BMI | -0.161 | 0.027 | 0.326 | <0.001 |
| HDL-C | 0.244 | 0.001 | -0.207 | 0.005 |
| GPT | -0.185 | 0.011 | 0.148 | 0.041 |
| TG | -0.058 | 0.431 | 0.116 | 0.114 |
| LDL-C | -0.012 | 0.868 | 0.106 | 0.149 |

Abbreviations: CES-D, Center for Epidemiologic Studies Depression Scale; HAM-A, Hamilton Anxiety Rating Scale; BMI, body mass index; HDL, high-density lipoprotein cholesterol; GPT, alanine amino transferase; TG, triglyceride; LDL, low-density lipoprotein cholesterol.
